# Systematic study of the iodinated rectal hydrogel spacer material discrepancy on accuracy of proton dosimetry

**DOI:** 10.1002/acm2.13774

**Published:** 2022-09-15

**Authors:** Hamed Hooshangnejad, Dong Han, Ziwei Feng, Liang Dong, Edward Sun, Kaifang Du, Kai Ding

**Affiliations:** ^1^ Department of Biomedical Engineering Johns Hopkins University School of Medicine Baltimore Maryland USA; ^2^ Department of Radiation Oncology and Molecular Radiation Sciences Johns Hopkins University School of Medicine Baltimore Maryland USA; ^3^ Department of Radiation Oncology The University of Maryland School of Medicine Baltimore Maryland USA; ^4^ Department of Electrical and Computer Engineering Johns Hopkins University School of Engineering Baltimore Maryland USA; ^5^ Department of Urology Renji Hospital Shanghai Jiao Tong University School of Medicine Shanghai China; ^6^ Brady Urological Institute Johns Hopkins University School of Medicine Baltimore Maryland USA; ^7^ Texas Center for Proton Therapy Irving TX USA

**Keywords:** hydrogel spacer iodination, neurovascular bundle, proton dose discrepancy, spacer‐enabled proton therapy

## Abstract

**Purpose:**

Iodination of rectal hydrogel spacer increases the computed tomography (CT) visibility. The effect of iodinated hydrogel spacer material on the accuracy of proton dosimetry has not been fully studied yet. We presented a systematic study to determine the effect of iodination on proton dosimetry accuracy during proton therapy (PT).

**Methods:**

PT plans were designed for 20 prostate cancer patients with rectal hydrogel spacer. Three variations of hydrogel density were considered. First, as the ground truth, the true elemental composition of hydrogel true material (TM), verified by our measurement of spacer stopping power ratio, was used for plan optimization and Monte Carlo dose calculation. The dose distribution was recalculated with (1) no material (NM) override based on the CT intensity of the iodinated spacer, and (2) the water material (WM) override, where spacer material was replaced by water. The plans were compared with the ground truth using the metrics of gamma index (GI) and dosimetric indices.

**Results:**

The iodination of hydrogel spacer affected the proton dose distribution with the NM scenario showing the most deviation from the ground truth. The iodination of spacer resulted in a notable increase in CT intensity and led to the treatment planning systems mistreating the iodinated spacer as a high‐density material. Among the structures adjacent to the target, neurovascular bundles showed the largest dose difference, up to 350 cGy or about 5% of the prescribed dose with NM. Compared to the WM scenario, dose distribution similarity and GI passing ratios were lower in the NM scenario.

**Conclusion:**

The inaccurate CT intensity‐based material for iodinated spacer resulted in errors in PT dose calculation. We found that the error was negligible if the iodinated spacer was replaced with water. Water density can be used as a clinically accessible and convenient alternative material override to true spacer material.

**Abbreviations and acronyms**
PT: proton therapy
RT: radiation therapy
TPS: treatment planning system
MRI: magnetic resonance imaging
HU: Hounsfield unit
OAR: organ‐at‐risk
ROI: region of interest
2D: two dimensional
3D: three dimensional
CT: computed tomography
CTV: clinical target volume
NM: no material
TM: true material
WM: water material
NVB: neurovascular bundle
GI: gamma index
DVH: dose–volume histogram
MC: Monte Carlo

## 1 INTRODUCTION

Prostate cancer is one of the most prevalent malignancies in men, and radiation therapy (RT) is shown to be an effective treatment for localized prostate cancer.^1^ Dose escalation improves the treatment outcome^2^, ^3^ but is limited due to radiosensitive organs‐at‐risk (OARs), namely, the rectum, bladder, and urethra. Depositing the nearly entire prescription dose to target without exit dose, proton therapy (PT) can decrease the low‐dose radiation to OARs.^4^, ^5^, ^6^, ^7^

Hydrogel spacer has been used in recent years to increase the separation between the rectal wall and prostate, enabling dose escalation to the target.^8^, ^9^, ^10^, ^11^, ^12^ Recent studies have shown that hydrogel spacer resulted in a significant reduction of rectal dose during PT.^13^, ^14^, ^15^, ^16^, ^17^, ^18^, ^19^, ^20^, ^21^, ^22^ However, the standard rectal hydrogel spacer is not visible on planning computed tomography (CT) and intraoperative cone‐beam CT that lack the adequate resolution and contrast to distinguish between the soft tissue (prostate and rectum) and hydrogel.^23^, ^24^, ^25^, ^26^, ^27^, ^28^, ^29^, ^30^ Thus, magnetic resonance imaging (MRI) is required to localize the hydrogel spacer, but anatomical changes, patient intolerance, MRI‐incompatible hardware, and the extra cost and time raise the need for an alternative spacer localization method. Iodination of the spacer by adding a small amount of iodine (*Z* = 53, K‐edge = 33.2 keV) is an effective solution to increase the spacer contrast on CT.^31^, ^32^

Accurate material density is critical for the calculation of proton stopping power and beam range. The miscalculation of safety margins will result in more severe consequences in PT compared to RT.^33^ Conventionally, the treatment planning systems (TPS) determine the proton stopping power based on the CT Hounsfield unit (HU). Because the iodine K‐edge is close to the mean energy of the planning CT, the iodinated hydrogel spacer has a high HU on planning CT. The high HU hydrogel spacer can be mistaken for a high‐density material with high proton stopping power due to the existence of high *Z* atomic number of iodine contrast. Here, we conducted a systematic study to determine the effect of the iodinated spacer material discrepancy on the accuracy of PT plans by comparing it to the true material (TM) composition of iodinated hydrogel spacer. We analyzed the similarity of the dose distribution using the clinically meaningful dosimetric and gamma indices and provide a recommendation on a clinically acceptable and practical planning strategy.

## 2 MATERIALS AND METHODS

### 2.1 Data preparation

20 cases of prostate cancer injected with hydrogel spacer (SpaceOAR System, Boston Scientific, Marlborough, MA) were used for this study. The clinical target volume (CTV) included the prostate and seminal vesicles. The scans were acquired with 2‐mm slice thickness, 120‐kVp, 200‐mA, and 50‐cm field of view as part of patients’ RT treatment, and contours were delineated by certified physicians.

### 2.2 Simulation of iodinated spacer

**FIGURE 1:**
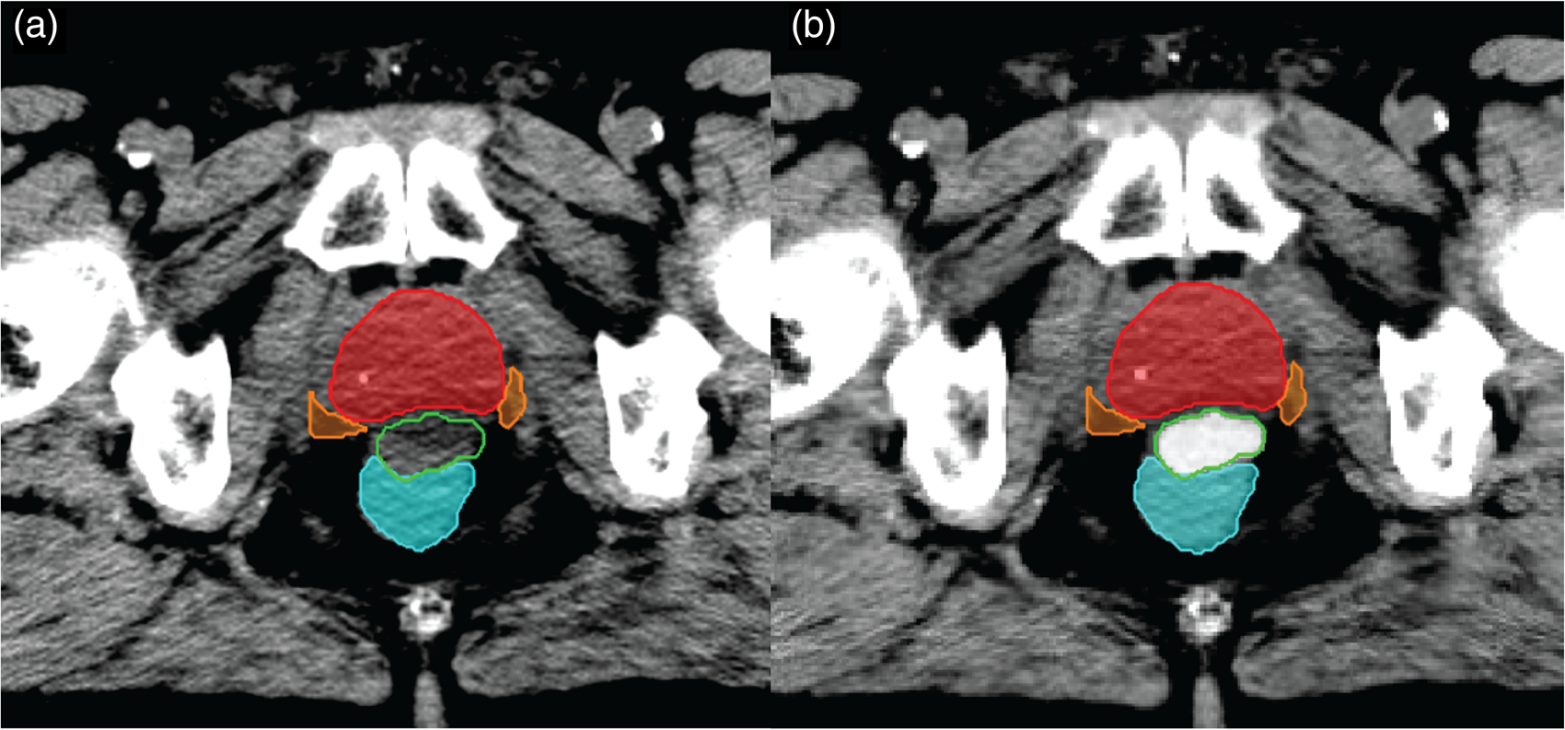
An illustration of spacer computed tomography (CT) intensity override: Part (a) shows the prostate (red), the rectum (cyan), the neurovascular bundle (orange), and the spacer (green). The original CT intensity is close to surrounding soft tissue making the localization of the spacer challenging. Part (b) shows the same CT slice with simulated iodinated spacer intensity.

The patients were originally injected with SpaceOAR that has roughly the same HU as soft tissue (average HU = 31). As a result, we simulated the iodinated hydrogel spacer (SpaceOAR Vue) by overriding the spacer HU with a normally distributed CT intensity with a mean value of 120. Spacer and the ROIs for RT planning were all delineated by a certified physician. The mean HU of 120 is based on our measurement using solid water phantom and patient data from our institution and reported values in previous studies.^32^, ^34^ 3D smoothing was applied to reduce the pixelation effect and simulate the realistic texture of hydrogel. All analyses were done using our MATLAB‐based in‐house image processing toolbox.^16^, ^35^, ^36^, ^37^, ^38^, ^39^, ^40^ Figure 1 shows a typical case of the original SpaceOAR (a) and simulated iodinated spacer (b). We used the CT scans with simulated iodinated spacer for the rest of the study.

### 2.3 Study design

We designed PT plans for three spacer material properties: (1) no material (NM) override, (2) water material (WM) override, and (3) hydrogel TM override as the ground truth. The scenarios differ from each other in the material property of the spacer used for proton dose calculation using RayStation TPS (RaySearch Laboratories, Stockholm, Sweden). For the NM scenario, TPS explicitly correlates the HU to the stopping power for dose calculation.^41^ As a result, the iodinated spacer with high HU will be treated as a high‐density structure.^42^ In the WM scenario, we manually overrode the material property of the spacer with water. For the TM scenario, we implemented the true elemental composition (88% water, 0.7% trilysine, 10.1% PEG, and 1.2% iodine by weight) of spacer in TPS using the information directly provided by the manufacturer for training purposes.

### 2.4 Stopping power verification

The SpaceOAR Vue material density was defined as 1.03 g/cm^3^ and effective *z* = 14.47, which is consistent with the previous study.^34^ However, in clinical practice, the stopping power ratio (SPR) is measured using a multilayer ion chamber to detect the shift of integral depth dose (IDD) due to the presence of the spacer. To confirm the material density of the spacer, we measured the SPR of the spacer under proton energies of 198.3 MeV. The spacer gel was prepared in 10‐ml powder vial that comes with the spacer prep toolkits. During the process of spacer gel preparation, the mixture of powder and liquid is stirred sufficiently to ensure the uniformity of the gel product. A single spot beam and multilayer ionization chamber from IBA (IBA Dosimetry, Schwarzenbruck, Germany) Giraffe are used for IDD measurement in water phantom. The result indicated that SPR is 1.03 relative to water, which confirms our theoretical calculation with the given elemental composition. Thus, the TM scenario was considered the ground truth.

### 2.5 Proton therapy planning

A total of 60 intensity‐modulated PT (IMPT) plans were designed. The IMPT plans were done using the RayStation TPS with Hitachi PROBEAT (Hitachi, Ltd., Tokyo, Japan) compact‐gantry pencil‐beam scanning system. Monte Carlo (MC) simulation with the uncertainty of 0.5% was used for the designed clinical‐grade plans (70 Gy in 28 fractions). Two lateral beams (90° and 270°) centered on the CTV centroid were used. CTV included the prostate and seminal vesicles. Following the clinical practice in our institution, the plans were designed with a 5‐mm and 3.5% setup and range uncertainties robustness optimization method. The clinical target objectives and OAR constraints were chosen according to our institution guideline: at least 95% of CTV volume receives 100% prescribed dose, bladder V70Gy < 10 cm^3^ (volume receiving 70 Gy), bladder V65Gy < 15%, bladder V61Gy < 25%, bladder V55Gy < 30%, bladder V44Gy < 50%, bladder V39Gy < 60%, rectum V70Gy < 10 cm^3^, rectum V65Gy < 10%, rectum V61Gy < 15%, rectum V53Gy < 30%, rectum V35Gy < 50%, right femur V39Gy < 5%, left femur V39 < 5%, penile bulb average dose <39Gy, and body $D_{0.03\;cm^{3}}$ (dose at 0.03‐cm^3^ volume) <74.9 Gy. Plans were optimized on the TM scenario, and the final dose distribution was recalculated for NM and WM scenarios to directly observe the material‐related deviation from the ground truth.

### 2.6 Dose distribution comparison

We quantified the deviation of NM and WM plans from the grand truth (TM) by comparing the 3D gamma index (GI) and dosimetric indices for each pair: the TM–NM pair and TM–WM pair.

#### 2.6.1 Gamma index analysis

We implemented a 3D GI calculation as described in Ju et al.^43^ The 3D GI matrices were obtained for three criteria: 1%/1 mm (PR11), 2%/2 mm (PR22), and 3%/3 mm (PR33). Same as the clinical practice in our institution, the passing ratio (PR) metric was calculated and defined as the ratio of the number of voxels with GI <1 over the total number of voxels, excluding the voxels with a dose less than 10% of the maximum dose. We considered a PR of more than 90% as insignificant dose difference in our study. In clinical practice, the 90% threshold is the clinical passing rate for PR22 and PR33.

#### 2.6.2 Dosimetric analysis

For selected ROIs, we calculated the following dose–volume histogram (DVH) metrics: (1) *D*_02_, *D*_05_, *D*_20_, *D*_50_, *D*_95_, and *D*_98_ defined as the minimum dose covering 2%, 5%, 20%, 50%, 95%, and 98%; (2) the mean dose of a structure is defined as the average dose over voxels of the structure; and (3) the equivalent uniform dose (EUD) is calculated using the following formula:

(1) $$EUD_{a}={\left(\sum_{i}n_{i}d_{i}^{a}\right)}^{\frac{1}{a}}$$

where $n_{i}$ is the fraction of structure with the dose $d_{i}$. *a* is an ROI‐dependent parameter for target structures, namely, prostate, seminal vesicles, and CTV a=−10, and OARs, namely, rectum, bladder, and neurovascular bundles (NVB), a=8.^44^, ^45^

### 2.7 Statistical analysis

**FIGURE 2:**
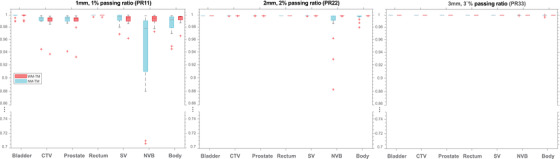
Boxplot of gamma index passing ratio (PR) showing the variation of gamma index (GI) PRs overall cases using different GI calculation criteria specific to each different structure and scenario

The differences were tested using the nonparametric permutation test (*n* = 1000) to circumvent the normality assumption. For the boxplots (Figures 2 and 4), the central mark indicates the median, and the top and bottom edges of the box indicate 75th and 25th percentiles, respectively. The horizontal mark on the whiskers shows the most extreme data (maximum and minimum) defined as 1.5 times of interquartile range away from the 75th and 25th percentiles, and “+” indicates the outliers.

## 3 RESULTS

### 3.1 Gamma index analysis results

Our measurement of the SPR of the spacer under proton energies of 198.3 MeV indicated that SPR is 1.03 relative to water, confirming our theoretical calculation with the given elemental composition. The TM scenario was considered the ground truth. Figure 2 shows the boxplot of the structure‐specific GI PRs. As seen, PR33 and PR22 were close to one for most structures except for the NVB. PR11, however, had a noticeable difference from one, and specifically for NVB, the PR11 got as low as 0.7 for the NM–TM scenario that is far below the clinical threshold of 0.9.

### 3.2 Dosimetric analysis

**FIGURE 3:**
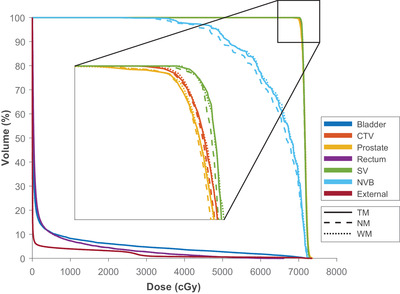
A typical case of the dose–volume histogram (DVH) graph, with a magnified part focusing on DVH curves of the high‐dose target volumes

The maximum deviation from ground truth was 372 cGy for the case of NVB‐*D*_98_ when NM override is used. Figure 3 shows a typical case of the DVH graphs. There is a stark difference between the three scenarios for NVB structure (Figure 3). The magnification on the target volumes revealed a minor decrease for the NM and WM scenarios compared to the ground truth. As in NM override, the spacer tends to be considered a high‐density material, and the NM scenario has the lowest DVH curves among the other scenarios.

**FIGURE 4:**
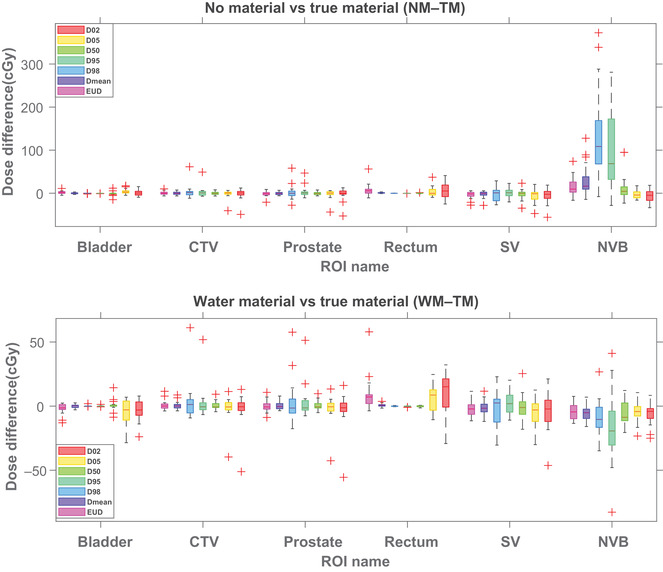
The differences between dosimetric indices between no material override and hydrogel true material (TM) (top figure) and water material override, and hydrogel TM (bottom figure) are broken down for each structure.

Figure 4 breaks down the dosimetric indices for the selected ROIs. As shown in the top part of Figure 4, the dose difference for NM–TM reached the maximum of more than 350 cGy in the case of NVB structure. Moreover, we found a statistically significant difference among EUD, *D*_mean_, *D*_50_, and *D*_95_ (*p*‐value <0.01). No specific pattern was found for the other ROIs. Finally, we found no strong relationship between spacer volume and the dose differences. The lower part of Figure 4 shows that the dosimetric indices tend to be lower for the WM–TM scenario with a maximum of less than 60‐cGy dose difference for NVB, with no statistically significant difference from zero.

## 4 DISCUSSION

In clinical practice, the material override is typically used for the probes and applicators that during the treatment delivery may not be present. However, for structures inside the body, the TPS assumes the material properties were accurately predefined on a voxel basis throughout the entire CT scan. This approach may lead to dose discrepancy if the actual and approximated CT intensity‐based material properties do not match. This study was designed to give an insight into the consequences of the spacer material discrepancy on proton dose distribution. Using a controlled setting in which the same CT scan and PT plan parameters were used, we exclusively study the error arising from spacer material variation. Our result showed that the use of incorrect material density resulted in inaccurate proton dose distribution, with the NM scenario showing a more adverse effect.

In agreement with our findings, the previous study has also shown that HU override is necessary for iodinated spacer.^34^ In their study, they suggest HU override as a possible solution to increase the accuracy of the proton dose calculation. However, TPSs like RayStation and Pinnacle (Philips Radiation Oncology Systems, Milpitas, CA) do not have an HU override option, but rather, they provide a material density override option. As a result, in our study, we introduced WM density override as an alternative solution. The main advantage of water density override is that it is predefined in all TPSs. Moreover, it can also be implemented by using the corresponding HU number that making it a more versatile solution to the iodinated spacer problem.

A considerably higher magnitude and statistically significant differences from the ground truth were observed for the NM scenario specifically for NVB. We believe this is due to the location of the NVB, as NVB and spacer are aligned from the lateral beams view. We also found an average decrease in dose‐to‐target volumes for the NM scenario (Figure 4). This underestimation of the proton dose due to the spacer's inaccurate higher stopping power may give rise to the TPS overcompensating for the target dose and, thus, increasing the OARs dose like NVB as shown in Figure 4 top part. As seen, all the differences from the ground truth were positive. In this study, we only used two lateral beams arrangement, in which the spacer, rectum, and target volumes can potentially be on the beam path. The effect of spacer material‐related dose discrepancy may be different for other beam arrangements. Hence, further studies to determine the effect of beam arrangement to find the optimized beam arrangement and spacer location^46^, ^47^ are warranted.

## 5 CONCLUSION

In this study, we presented a detailed analysis of the effect of iodinated spacer material discrepancy on proton dose distribution. We found statistically significant dose inaccuracy, for NM override, which was highly variable with patient anatomy, spacer location, and beam arrangement. WM override, on the other hand, showed high agreement with the ground truth. Accordingly, we argue that the WM is a clinically acceptable and convenient overriding strategy for iodinated spacers during plan optimization and dose calculation.

## AUTHOR CONTRIBUTIONS

Hamed Hooshangnejad, Dong Han, and Kai Ding designed the study and prepared the manuscript. Hamed Hooshangnejad, Dong Han, Edward Sun, and Kai Ding contributed to data analysis and interpretation. Hamed Hooshangnejad, Dong Han, Ziwei Feng, and Kai Ding participated in collecting data. All authors contributed to the article and approved the submitted version.

## CONFLICT OF INTEREST

The authors declare no conflict of interest.
